# Molecular, Phenotypic Aspects and Therapeutic Horizons of Rare Genetic Bone Disorders

**DOI:** 10.1155/2014/670842

**Published:** 2014-10-22

**Authors:** Taha Faruqi, Naveen Dhawan, Jaya Bahl, Vineet Gupta, Shivani Vohra, Khin Tu, Samir M. Abdelmagid

**Affiliations:** ^1^Nova Southeastern University Health Sciences Division, Fort-Lauderdale-Davie, FL 33314, USA; ^2^Florida International University (FIU), Miami, FL 33174, USA; ^3^Department of Medicine, University of California San Diego (UCSD), 200 West Arbor Drive, MC 8485, San Diego, CA 92103, USA; ^4^University of Pennsylvania School of Dental Medicine, Philadelphia, PA 19104, USA; ^5^Northeast Ohio Medical University (NEOMED) School of Medicine, Rootstown, OH 44272, USA

## Abstract

A rare disease afflicts less than 200,000 individuals, according to the National Organization for Rare Diseases (NORD) of the United States. Over 6,000 rare disorders affect approximately 1 in 10 Americans. Rare genetic bone disorders remain the major causes of disability in US patients. These rare bone disorders also represent a therapeutic challenge for clinicians, due to lack of understanding of underlying mechanisms. This systematic review explored current literature on therapeutic directions for the following rare genetic bone disorders: fibrous dysplasia, Gorham-Stout syndrome, fibrodysplasia ossificans progressiva, melorheostosis, multiple hereditary exostosis, osteogenesis imperfecta, craniometaphyseal dysplasia, achondroplasia, and hypophosphatasia. The disease mechanisms of Gorham-Stout disease, melorheostosis, and multiple hereditary exostosis are not fully elucidated. Inhibitors of the ACVR1/ALK2 pathway may serve as possible therapeutic intervention for FOP. The use of bisphosphonates and IL-6 inhibitors has been explored to be useful in the treatment of fibrous dysplasia, but more research is warranted. Cell therapy, bisphosphonate polytherapy, and human growth hormone may avert the pathology in osteogenesis imperfecta, but further studies are needed. There are still no current effective treatments for these bone disorders; however, significant promising advances in therapeutic modalities were developed that will limit patient suffering and treat their skeletal disabilities.

## 1. Introduction

In the spectrum of orthopaedic diseases, rare genetic bone disorders are often ignored as major diseases such as osteoporosis generally attract more research funding and attention from the research community. A rare disease is defined as one affecting less than 200,000 individuals, according to the US National Organization of Rare Diseases (NORD). Rare bone disorders remain a serious problem in orthopaedics and result in significant morbidity and mortality in patients around the world.

Often a primary problem with rare bone diseases remains to be a lack of understanding of the underlying mechanism. Yet, in recent years many advances have occurred that are promising for the prospect of finding cures. In 2006, the gene for fibrodysplasia ossificans progressiva (FOP) was identified by researchers at the University of Pennsylvania, marking a significant milestone in the understanding of this disease. Prior to this, its etiology remained elusive. While this does not in and of itself translate to a cure, the discovery provides direction for researchers to investigate possible points of disruption of the basic pathway of FOP. Yet, other rare disorders still remain mysteries.

This review summarizes the most current trends in the search for therapeutic interventions for nine rare bone disorders: fibrous dysplasia, Gorham-Stout syndrome, fibrodysplasia ossificans progressiva, melorheostosis, multiple hereditary exostosis, osteogenesis imperfecta, and craniometaphyseal dysplasia.

## 2. Fibrous Dysplasia

Fibrous dysplasia (FD) is a rare bone disease characterized by replacement of the medullary cavity with fibrous tissue. Any region of the skeleton can be affected by FD, where the most common areas involved include facial bones, the tibia, femur, and the ribs [1]. Several forms of FD exist. The monostotic form of FD is limited to one bone, whereas the polyostotic form is manifest in multiple bones [2]. McCune-Albright syndrome is another variant of FD and, in addition to bone involvement, is associated with endocrine dysfunctions such as Cushing syndrome, hyperthyroidism, and acromegaly [1, 2]. FD causes chronic pain in patients due to bone overgrowth. Other long term problems include bony deformities, unequal limb lengths, and diminished bone strength leading to a high risk of fractures.

FD displays no predilection for either gender. The monostotic form is more prevalent than the polyostotic form, with the variants occurring at a ratio of 7 : 3, respectively [3]. The monostotic form classically occurs in individuals in their 20s to 30s whereas the polyostotic form is usually seen in children. Polyostotic FD usually enters dormancy at the onset of puberty, but pregnancy may result in reactivation of the disease [1].

FD results of mutations in the guanine nucleotide binding, alpha stimulating (GNAS) complex locus, located on chromosome 20 [4]. The mutations occur postzygotically and lead to constitutive activation of G*α*_s_, resulting in stimulation of the Wnt/*β*-catenin signaling pathway [4, 5]. Mutation activation of G*α*_s_ subunit leads to high levels of cyclic adenosine monophosphate (cAMP) levels that mediate the downstream functions in the affected cells. In particular, the transcription factors cFos and cJun and the cytokine IL-6 are upregulated in osteoclasts, resulting in excessive bone resorption and dysplastic fibrous growth [1, 6].

Recent study showed that transgenic mice with constitutive expression of the G*α*_s_ subunit developed an inherited pathologically replication of human FD. The characteristic FD lesions in mice developed only in postnatal life as in human FD [6]. In the affected bone, the lesions develop through a sequence of three consecutive stages: a primary modeling phase characterized by excess medullary bone formation; a secondary phase, with excess, inappropriate remodeling; and a tertiary phase of fibrous dysplastic in the marrow cavity that replicates the human bone pathology in mice of more than 1 year old [6, 7].

X-ray diagnostic features of FD are a characteristic hazy bone lesion (ground glass). For most parts, this radiologic entity is sufficient for the initial diagnosis of the disease. However, in patients where metastasis may pose a viable concern, a PET/CT may be considered. However, Su et al. concluded that this alone may not be enough [8]. They conducted F-fluoro-2-deoxy-glucose positron emission tomography (F-FDG PET/CT) on a female patient in whom breast cancer recurrence was suspected. FD was an incidental finding on PET/CT. However, they noted that the dysplastic lesion mimicked metastasis. MRI proved to be a useful modality in differentiating FD from metastasis. Other novel approaches of detecting the disease are also being pursued. Tabareau-Delalande et al. [9] demonstrated that GNAS mutations are specific for fibrous dysplasia among other fibroossifying lesions. Thus, DNA markers for the GNAS mutation may provide an alternate means of diagnosing the disease in more complicated cases of FD.

In the present, there is no cure for FD and the management is composed largely of reduction of pain, preventing further degeneration of bone, and surgical intervention to reshape and restore the functionality of the affected bone. A current approach that aims at both strengthening bone and reducing pain is bisphosphonate therapy. Mäkitie et al. [10] administered bisphosphonates intravenously in a patient with mandibular FD. The therapeutic approach resulted in rapid reduction of pain, stabilized turnover of bone, and even proved to be cosmetically beneficial. In patients that are nonresponsive to bisphosphonates, Chapurlat et al. [11] suggested the use of IL-6 inhibitors such as tocilizumab, a monoclonal antibody used to treat rheumatoid arthritis (RA). A study investigating the effect of tocilizumab on systemic bone resorption through tracking serum cross-linked C-terminal telopeptide of type I collagen (CTX and ICTP) revealed a significant decrease in bone resorption with the therapy [12]. Therefore, this approach could also be useful in preventing the bone resorption seen in FD.

**Figure 1:**
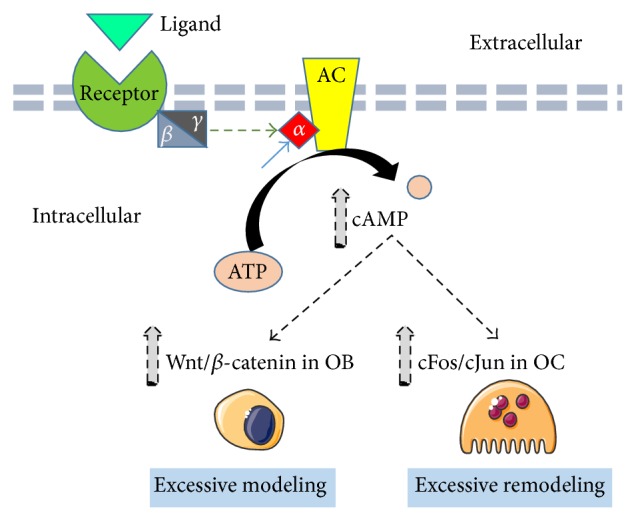
Schematic diagram of the pathogenesis of FD: mutation of the *α* subunit in GNAS (blue arrow) results in autonomous activation of adenylate cyclase (AC) and increased cAMP levels. Cyclic AMP stimulates Wnt/*β*-catenin signaling in osteoblasts leading to excessive bone formation. In addition, cAMP activates cJun and cFos of AP-1 complex in osteoclasts resulting in excessive bone remodeling.

**Figure 2:**
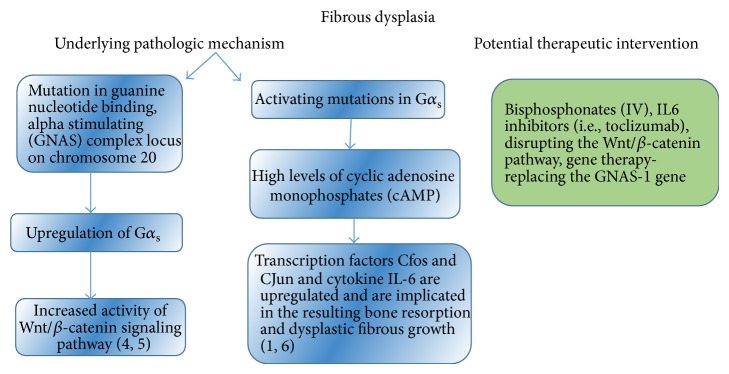
Summary of the pathological mechanisms underlying FD and potential therapeutic strategies that may be pursued.

Several potential therapeutic interventions may be employed (Figure 1). A possible therapeutic strategy to be pursued in the future could be targeting the Wnt/*β*-catenin pathway. If the Wnt signaling pathway is halted, *β*-catenin will not accumulate within the cell since it is marked for ubiquitination by casein kinase 1*α* (CK1*α*), protein phosphatase 2A (PP2A) adenomatosis polyposis coli (APC), Axin, and glycogen synthase kinase 3 (GSK3) [13]. Ubiquitination of *β*-catenin would lead to its proteasomal degradation, thus preventing it from eliciting a cellular response contributing to FD. Therefore, if Wnt proteins can be selectively bound by ligand analogs and inactivated, the tumorigenic fibrous growth will be diminished (Figure 2).

## 3. Gorham-Stout Disease

Gorham's disease (GD), also known as vanishing bone disease, is a rare genetic disorder characterized by bone resorption and localized lymphangiogenic proliferation [14]. This lymphatic and vascular proliferation within bone is thought to aid in osteolysis. GD shows no preference for gender or race and occurs more often in children and young adults. Although GD manifests itself as a monostotic or polyostotic disease, it more commonly involves the flat bones that form by intermembranous ossification [15].

Diagnosis of GD is challenging; it is often a diagnosis of exclusion. Other differentials such as endocrinopathies, malignancies, and immunologic, infectious, and metabolic etiologies need to be ruled out before a diagnosis of GD can be made [15, 16].

A study conducted by Venkatramani et al. [17] revealed insights about GD manifestations. Of the eight patients (median age at diagnosis was 11.5 years) who were part of the study, seven presented with lymphangiomatous lesions in the soft tissues adjacent to the involved bone. This finding is particularly interesting since Bruch-Gerharz et al. [18] also found that skin and soft tissues adjacent to the bone lesions have remarkable lymphatic vascular malformations. Furthermore, the skin and soft tissue involvement preceded bone osteolysis by several years. Therefore, one can conclude that the lymphatic vascular malformations presenting in GD can potentially serve as an early diagnostic sign. Bruch-Gerharz et al. also demonstrated that magnetic resonance imaging (MRI) was essential in characterizing the extent of GD progression by tracking lymphatic malformation in tissues [18].

**Figure 3:**
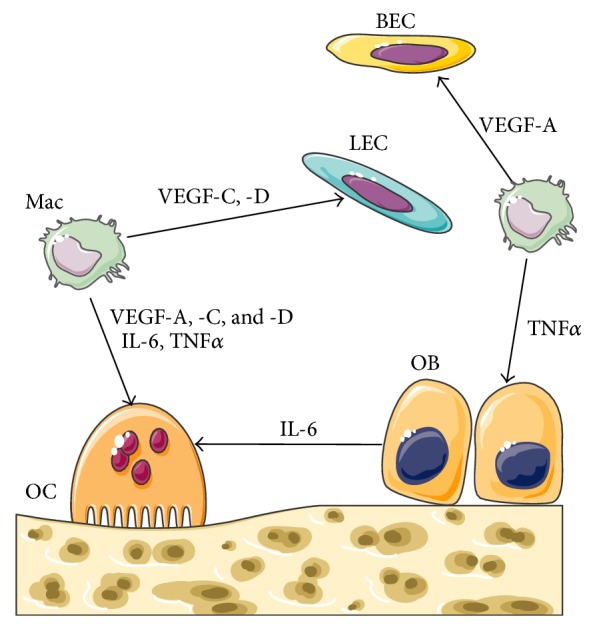
Schematic diagram of the pathogenesis of GSD. Lymphatic and blood endothelial cells (LECs), BECs, and macrophages (Mac) secrete TNF*α* that stimulate OB to release IL-6. Mac produces VEGF-C and -D that stimulate proliferation of LECs and BECs. Mac also produces VEGF-A, -C, and -D and IL-6 that directly stimulate osteoclast-mediated bone resorption.

The pathogenesis of GD is not well understood and therefore not many therapeutic modalities are currently available. Recent study showed that lymphatic endothelial cells (LECs) and blood endothelial cells (BECs) in addition to macrophages secrete TNF*α* and IL-6 that stimulate osteoclast formation with excessive osteolysis [19]. Macrophages produce VEGF-C and -D that stimulate proliferation of LECs and BECs. Moreover, macrophages produce VEGF-A, -C, and -D and IL-6 that directly stimulate osteoclast differentiation [20] (Figure 3). Furthermore, TNF*α* secreted by LECs and macrophages inhibits osteoblast differentiation and new bone formation [21]. Devlin et al. [22] demonstrated that the serum from a patient with GD caused increased proliferation of osteoclast-like multinucleated cells when cultured with normal human bone marrow. Furthermore, the levels of IL-6 were significantly higher in the serum of GD patients. This suggests that bone resorption observed in GD could be a direct result of increased multinucleated cell activity due to increased IL-6 levels. Therefore, local inhibition of IL-6 production or administration of a drug such as tocilizumab will be beneficial.

**Figure 4:**
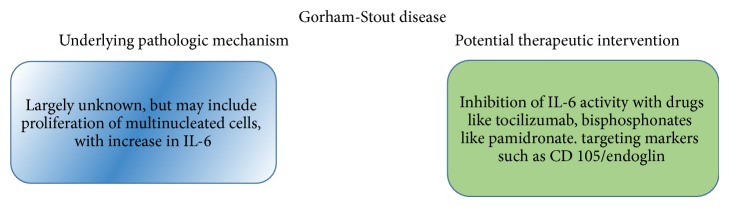
Pathogenesis of GD and potential therapeutic interventions.

Today, there are no set guidelines for the treatment and management of GD. To prevent the production of IL-6 by proliferating vasculature, radiation therapy, and chemotherapy with interferon *α*-2b is commonly employed [23], although it is contraindicated in growing children. Different treatment modalities that include surgical resection, arthroplasty, calcitonin, calcium, and vitamin D have been utilized and the results are variable. Bone grafts have also been used, with a debatable successful rate. Hirayama et al. [16] reported that, despite the use of a bone graft, GD recurred in the grafted bone. In a revealing case described by Hammer et al. [24], clinical improvement followed by stabilization of the disease occurred solely after use of low-dose pamidronate therapy. To our knowledge, this is the only known case of a bisphosphonate monotherapy leading to remission of GD (Figure 4).

Other efforts include the identification of diagnostic markers of GD. In a study conducted by Franchi et al. [25], CD105/endoglin, a marker for vascular endothelial cells, was used to assess the nature of the endothelial cells proliferating in GD. CD105 expression was found to be significantly higher in GD vessels compared to those found in osseous hemangioma (% positive was 58.9 versus 17.2, resp.). Therefore, this marker may offer a potential means of diagnosing patients with GD.

## 4. Fibrodysplasia Ossificans Progressiva

Fibrodysplasia ossificans progressiva (FOP) is a rare devastating autosomal dominant disease that is characterized by heterotrophic ossification (HO) in the soft tissues following a simple injury [26]. The disease affects 1 in 2 million individuals [27]. There are currently about 700 known cases around the world. FOP displays no predilection for gender, race, or geographic location [28]. Although episodic flare-ups occur in FOP, the damage is cumulative, leading to increasing disability. Individuals with FOP display no abnormality at birth, with the exception of congenital great toe malformations [27]. Painful transformation of soft connective tissue into bone begins in the first decade of life [29]. Surgical intervention leads to a sever rebound response marked by rapid bone growth [28].

HO in FOP is seen initially in the cranial, dorsal, axial, and proximal regions of the body and then later occurs in caudal, ventral, and distal regions. Since there are episodic flare-ups, the disease progression may vary and not follow the previous order in all cases. Skeletal muscles are also involved in the ossification process; however, smooth muscle and cardiac muscle are spared [28]. Kaplan et al. [30] conducted a study to determine the cause of death and lifespan of individuals with FOP. The most common cause of death in FOP was cardiorespiratory failure as a result of thoracic insufficiency syndrome, and the median lifespan of the 371 individuals in the international FOP community was 56 years.

The diagnosis of FOP can be made by the association of progressive ossifying soft tissue swellings and great toe malformations [31]. This association is not often made by clinicians and thus FOP is frequently missed. The affected individuals are often exposed to unwarranted trauma due to unneeded biopsies of the soft tissue swellings, thereby leading to further exacerbation of the disease.

**Figure 5:**
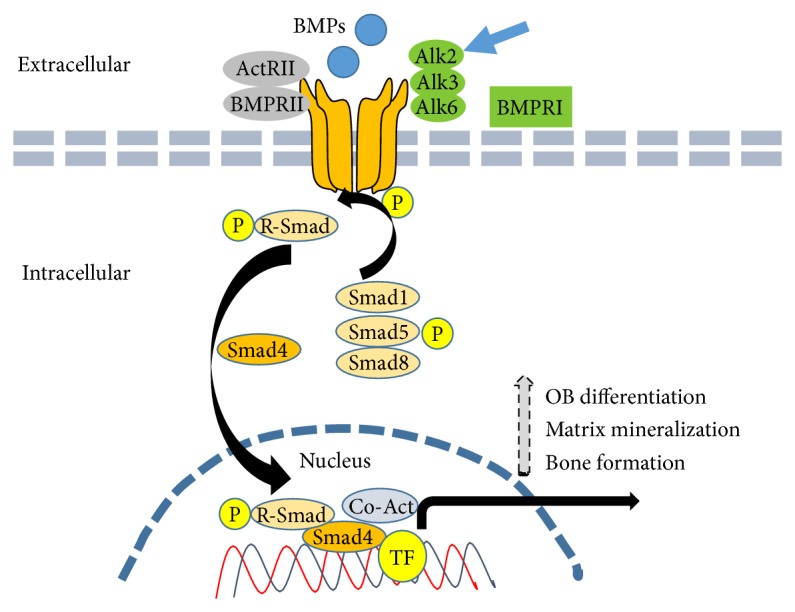
Schematic diagram of the pathogenesis of FOB: mutation of the Alk2 subunit (blue arrow) of BMP receptor I leads to constitutive phosphorylation of the downstream regulated-smad1, -5, and -8 that associate with smad4. Multimeric smad complex translocates to the nucleus and positively regulates several transcription factors responsible for osteoblast differentiation and bone formation.

There is no current cure for FOP. The current management of FOP is early diagnosis, preventing iatrogenic trauma, and alleviating pain during episodic flare-ups. Several studies have indicated that FOP is associated with the bone morphogenetic protein (BMP) signaling pathway. BMPs are responsible for the stimulation of bone formation through binding to the activin receptor type 1 (encoded by the AVCR1 gene receptor), a BMP type 1 receptor. Thus, in 2006 Kaplan et al. [26] identified a mutation in activin receptor IA/activin-like kinase 2 (AVCR1/ALK2) in all patients presenting with FOP (Figure 5). DNA sequencing displayed the occurrence of missense mutation in the glycine-serine activation domain in individuals with FOP. Not all FOP cases are caused by the common mutation, as there are several FOP variants with varying phenotypes. Importantly, Chakkalakal et al. [32] further elucidated the mechanism of FOP using a FOP knock-in mouse model. Thus, FOP results from a mutation in the gene ACVR1/ALK2, which causes the amino acid histidine to be substituted in place of arginine at the 206 codon. Due to the discovery of this highly specific mutation in the FOP gene, therapeutic modalities can now be aimed at blocking the AVCR1/ALK2 pathway. Thus, the identification of factors that are a part of or that aid the BMP signaling pathway has been the focus of recent studies. Mao et al. suggested the potential role of matrix metalloproteinase-10 (MMP-10) in the HO of muscle in FOP patients. They showed that MMP-10 stimulated myoblast differentiation into osteoblasts through the interactions with BMP pathway [33]. Thus, MMP-10 may serve as a potential therapeutic target. Giacopelli et al. [34] recently reported a significant finding that transcription factors including Egr-1, Egr-2, ZBTB7A/LRF, Hey1, and Sp1 are responsible for the regulation of the ACVR1 promoter through binding to the −762/−308 region. Furthermore, additional studies have shown that miR-148a may be a critical mediatory agent of ACVR1 [35, 36]. Thus, disruption of the pathway through blocking or slowing down any of these transcription factors presents the most promising form of potential therapy to date.

**Figure 6:**
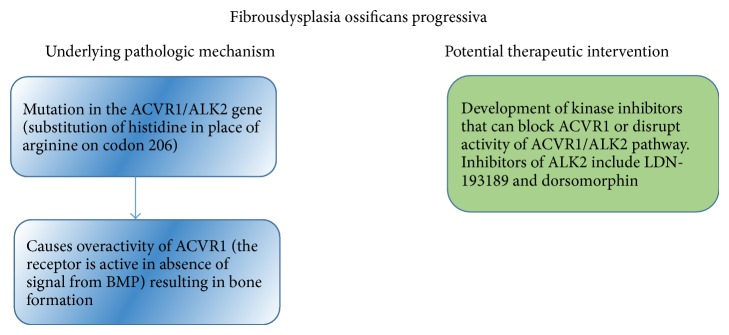
Summary of the pathogenesis of FOP and potential therapeutic interventions.

Importantly, while inhibitors of ALK2 including LDN-193189 and dorsomorphin are effective in reducing ALK2 activity, they also block the activity of another BMP receptor, BMPR1 (ALK3) activity [37]. Thus, any viable therapeutic intervention would be one that blocks the hyperactivity of ALK2 without impacting the other kinases in the pathway [33]. Kaplan et al. were able to identify siRNAs which target the ALK2 causing pathology while the normal ALK2 remained unaffected [37, 38]. Thus, siRNAs from FOP patients have been utilized to retain normal activity of BMP [37, 38]. Kaplan et al. [38] demonstrated selective suppression of mutated ACVR1 by utilizing ASP-RNAi (allele-specific RNA interference) techniques. This study showed a promising glimpse of the possibility of shutting down ACVR1 activity. Yet, further work is needed to develop an effective regimen of ACVR1 suppression in humans.  Figure 6 summarizes the pathogenesis and possible therapeutic strategies that may target FOP.

## 5. Melorheostosis

Melorheostosis is a rare genetic bone disease of unknown etiology in which patients exhibit bone dysplasia marked with benign sclerosis [39]. The disease has no predilection for gender and occurs sporadically. Scleroderma of the skin overlying the affected bone, vascular malformations, and soft tissue masses have also been reported [40]. Spinal sensory nerves are commonly involved [41] and the sclerosis is usually unilateral. The disease can be monostotic and polyostotic or only involve one limb (monomelic) [42]. Involvement of the lower limbs is more commonly seen whereas skull involvement is rare [42]. Histological analysis reveals thickening of the cortical bone that is comprised of mature lamellar and woven bone with adjacent fibrocartilage surrounding coronoid islands [43, 44].

The classic radiologic appearance of melorheostosis is “flowing hyperostosis” similar to hardened wax dripped on the side of a candle [41]. As such, upon classic presentation of the disease, diagnosis can be made by X-ray studies followed by increased uptake of radionuclide [41, 45]. The diagnosis can be confirmed by MRI and CT by detecting hyperostosis. Furthermore, MRI can also be used to determine the degree of soft tissue involvement [41]. However, Hollick et al. [45] noted that a milder presentation of melorheostosis may be more challenging to diagnose due to periosteal osteosarcoma and myositis ossificans competing as viable differentials.

**Figure 7:**
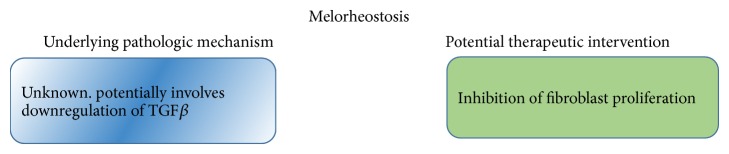
Pathogenesis and potential therapeutic interventions of melorheostosis.

There is no treatment for melorheostosis, although several potential therapeutic modalities have been suggested (Figure 7). Current management is highly individualized and is based on the severity of the disease, areas of skeletal involvement, and symptoms experienced by the patient. Surgical treatment is undertaken when an adverse or life threatening complication needs to be avoided. Zeiller et al. [41] performed cervicothoracic decompressive laminectomy to alleviate the worsening neurologic condition in their patients. A follow-up examination conducted six months after the surgery revealed symptomatic improvement of the disease. In another case, Moulder and Marsh [46] were successfully able to treat melorheostosis by total knee arthroplasty. Recently, Hollick et al. [45] were able to achieve a significant reduction of the lesions in melorheostosis with the associated symptoms by a single 5 mg infusion of zoledronic acid administered over a duration of 30 minutes. A follow-up conducted eighteen months after the initial therapy revealed an asymptomatic patient with no further need for treatment.

Hellemans et al. [47] initially linked the etiology of melorheostosis (along with osteopoikilosis and Buschke-Ollendorff syndrome) to mutations in the LEMD3 gene. However, in a later study conducted by Hellemans et al. [48], no LEMD3 mutations were identified in patients presenting solely with sporadic melorheostosis. Due to this discovery, the etiology of melorheostosis remains unknown.

Kim et al. [49] found that downregulation of adhesion proteins that regulate osteoblasts, particularly TGF-*β* induced gene product, occurs in melorheostosis. They hypothesized that this may be the cause of the presenting hyperostosis and soft tissue abnormalities. Examining the TGF-*β* pathway may provide some clues of the mechanism of melorheostosis. Endo et al. [50] displayed the fact that soft tissue and skin changes occurred due to increased secretion of collagen from fibroblasts. In addition, they proposed that hyperostosis may be responsible for stimulation of fibroblastic secretion. Therefore, inhibition of fibroblast proliferation may lead to an improvement in the soft tissue and skin manifestations of the disease.

## 6. Multiple Hereditary Exostosis

Multiple hereditary exostosis (MHE) is a genetic disorder marked by multiple cartilage-capped boney protuberances (osteochondromas) of the axial skeleton presenting usually before twelve years of age. The usual presentation is unequal limb lengths, reduced range of motion, and osteoarthritis [51]. Joints of the upper and lower limb are commonly affected, particularly the humerus, distal femur, and tibia; however, any bone might also be affected [52].

Diagnosis is made, as outlined by Wuyts and Van Hul [53], primarily using radiologic studies. The characteristic radiographic presentation of MHE is an uninterrupted continuation of the bone cortex into the osteochondroma. Additionally, a family history remarkable for MHE also aids in diagnosis [53].

*Pathogenesis of MHE*. The genetic basis of MHE has been identified due to mutations in the exostosin-1, EXT1, and EXT2 genes. These genes are involved in heparan sulfate (HS) chain elongation in the Golgi apparatus [54]. Multiple studies have found a more severe disease presentation in individuals with EXT1 mutations versus those with EXT2 mutations [55, 56]. Recent study showed that inactivation of EXT1 in mouse chondrocytes leads to the development of osteochondroma with characteristic bone deformities that is almost identical to human MHE [57]. It has been reported that EXT1 function is required for maintenance of normal levels of bone morphogenetic protein (BMP) and Wnt, as well as their target genes [58]. Another study indicated that loss of *β*-catenin expression (downstream target of BMP) in chondrocytes induces periosteal chondroma-like masses, resulting in the cartilage cap in osteochondromas [59].

Since the mutation is known, genetic testing is also currently available for diagnosis of MHE [53]. A novel method of diagnosing MHE has been proposed by Anower-E-Khuda et al. [60]. In their study, they compared HS and chondroitin sulfate (CS) from the serum of MHE patients and healthy individuals. They found that HS was significantly less in the serum of MHE patients and the HS/CS ratios were nearly half those of healthy individuals. Therefore, it was suggested that the HS/CS ratios may be utilized as a diagnostic predictor of MHE.

After diagnosis of MHE, the locations of the lesions, associated symptoms, and any structural deformities and functional limitations need to be documented. If the condition is asymptomatic, no therapy is indicated [53]. Surgeries, when performed, are usually done to limit the presenting symptoms or correct bone defects [61]. Due to undergrowth of the fibula, valgus deformities of the knee and ankle are usually seen [62]. In the upper extremity, the ulna is usually involved in causing radial deformities such as radial head dislocation and radial bowing to occur [63]. Surgical intervention is used in all of these cases.

A serious complication of MHE is malignant transformation into chondrosarcoma [64]. The risk for malignant transformation was previously reported to be 0.6 to 2.8% [65]. In contrast to this, Kivioja et al. [51] determined higher risk for transformation to chondrosarcoma at 8.3% in six generations of a family with prevalent MHE. Other literatures, however, reported the risk of malignant transformation as very low [66]. A relatively rare and unique complication that Khan et al. [67] reported in MHE patients was lower extremity ischemia due to popliteal artery occlusion.

**Figure 8:**
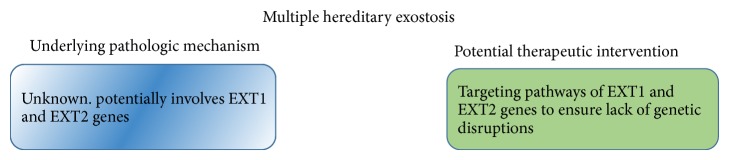
Pathogenesis of MHE and potential therapeutic interventions.

Currently, there is no cure for MHE. Although the genetic mutations have been identified, the genetic pathogenesis and particular signaling pathways that lead to the manifestation of the disease remain unknown (Figure 8). If the signaling pathways of EXT1 and EXT2 can be understood, molecular biology can potentially be utilized to alleviate the genetic disturbances due to lack of functional EXT1 and EXT2 genes.

## 7. Osteogenesis Imperfecta

Osteogenesis imperfecta (OI) is a rare genetic bone disease, characterized by the high incidence of fractures with or without minor trauma [68]. Hearing loss is a more commonly observed symptom of OI in older patients. Other classic features, seen in patients with OI, include blue sclerae and triangular facies.

*Pathogenesis of OI*. Type I collagen is an extracellular matrix protein, mainly found in bone and skin [69]. Two important steps of posttranslational modifications occur: first, hydroxylation of lysine and proline residues that occurs and conveys stability of the collagen triple helix; second, 3-hydroxylation of a proline residue that occurs in the *α*-one chain of type 1 collagen (COL1A1) at position 986 (P986) [69]. In autosomal dominant OI, mutations occur in COL1A1 and COL1A2 that preclude the right folding of type I collagen into proper triple helical structure [69]. Autosomal recessive lethal OI is caused by mutations in cartilage-associated protein CRTAP and prolyl-3-hydroxylase-1 (P3H1, encoded by LEPRE1 gene) which lead to decreased 3-hydroxylation of P986 in type I collagen's *α*-one chain. In both cases, overmodification of type I collagen is noted [69].

A knock-in mouse model for moderately sever OI has been generated [67, 70]. Characterization of the cellular contribution into the brittle bone disease showed a decrease of the cortical and trabecular bone before and after puberty resulting in 50% reduction of the bone mass compared to the wild type [70]. Although osteoblasts matrix production was greatly diminished, osteoclast number and activity were increased in the OI mouse compared to the wild type [70]. The study concluded uncoupling between osteoblasts and osteoclasts in brittle bone disease, perhaps due to higher expression of RANK receptors on osteoclast precursors [70]. This cellular imbalance results in decreased bone formation with aging. Interruption of the stimulus that increases osteoclast precursors may leads to new therapeutic modalities for OI. Interestingly, separate study reported the therapeutic benefits of RANKL inhibitors (RANK-Fc) and bisphosphonates in treatment of OI via increased number of bone trabeculae that reduce the incidence of fracture risks [71].

Diagnosis of OI is made based on a history of fractures, family history remarkable for OI, radiographic studies that reveal multiple fractures at different stages of healing, and genetic testing for mutations in COL1A1 and COL1A2. Additionally, biochemical testing of type I collagen may also be conducted. The biochemical testing consists of culturing dermal fibroblasts and analyzing the structure and quantity of the type I collagen produced. Four types of COL1A1 and COL1A2 related OI have been identified (I, II, III, and IV), and biochemical testing has a high sensitivity for detecting these four types of OI [68]. Although the sensitivity of biochemical analysis and genetic testing is comparable, genetic testing is still the recommended first line test for confirmation of OI [72].

Management of the disease is based on the degree of disease progression. Caregivers and parents are advised to handle OI patients safely, since they are susceptible to fractures. As such, management is primarily supportive [73]. Symptomatic surgical interventions include bracing of limbs, stabilization of joints, and reduction of boney deformities [73].

Cases have been reported in which bisphosphonates have been used in an attempt to alter the disease course. Phillipi et al. [74] elaborated the use of bisphosphonates to treat OI. The study indicated that although bone mineral density (BMD) and adult height of patients increased with bisphosphonate therapy, fracture incidence did not decline. This was further confirmed in the study conducted by Sakkers et al. [75], in which the researchers were unable to determine whether the use of olpadronate was able to alter the progression of OI.

**Figure 9:**
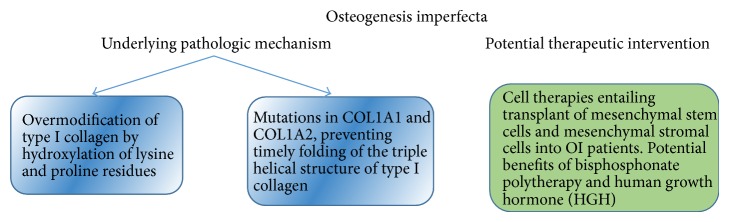
OI pathogenesis and potential therapeutic interventions.

Though there is no cure for OI, several therapies are being investigated (Figure 9). A study conducted by Antoniazzi et al. [76] investigated the effects of human growth hormone (HGH) and bisphosphonate polytherapy. The use of growth hormone was correlated with increased BMD and linear growth. Marini et al. [77] conducted a study that yielded similar results. Recently, Otsuru et al. [78] transplanted mesenchymal stem cells and mesenchymal stromal cells into patients with OI. The cell therapies proved to be very effective in this pilot clinical trial. This holds promise for a potential cure for OI in the near future.

## 8. Craniometaphyseal Dysplasia

Craniometaphyseal dysplasia (CMD) is an extremely rare genetic bone disorder characterized by overgrowth and progressive sclerosis of the craniofacial bones (cranium) and flaring of the metaphyseal plates of femurs (metaphyseal dysplasia) [79, 80]. The lifespan of patients diagnosed with craniometaphyseal dysplasia is normal, except in the most severe cases [81].

The characteristic bone outgrowth in the skull causes many of the symptoms and signs, seen in patients suffering from craniometaphyseal dysplasia. Affected individuals will typically have distinguishing facial features such as thickening of the cranial bones, prominent forehead, paranasal bossing, wide nasal bridge, wide-set eyes (hypertelorism), and a prominent jaw [82]. Infants affected by CMD will have excessive new bone formation (hyperostosis) in their jaw, resulting in delayed teething (dentition) or failure of teeth eruption [83, 84]. These infants with CMD may also have breathing or feeding problems due to narrow nasal passages. In the most severe cases, abnormal bone outgrowth can compress the cranial nerves emerging from the brain leading to paralyzed facial muscles (facial nerve palsy), blindness, or deafness [82, 84].

Craniometaphyseal dysplasia has two ways of inheritance, the autosomal dominant CMD that is typically more severe than the autosomal recessive form. In most cases this condition is inherited in an autosomal dominant pattern, which means a mutation in one gene copy in each cell is sufficient to cause the CMD disorder [81, 85, 86]. As craniometaphyseal dysplasia runs in families, patients with autosomal dominant CMD typically have one parent who also has the condition. Less often, cases result from new mutations in the gene and occur in people with no history of the disorder in their family. Rarely, craniometaphyseal dysplasia is suspected to have autosomal recessive inheritance when unaffected parents have more than one child with the condition. Autosomal recessive disorders are caused by mutations in both copies of a gene in each cell. The parents of an individual with an autosomal recessive condition each carry one copy of a mutated gene, but they typically do not show signs and symptoms of the disorder [87].

**Figure 10:**
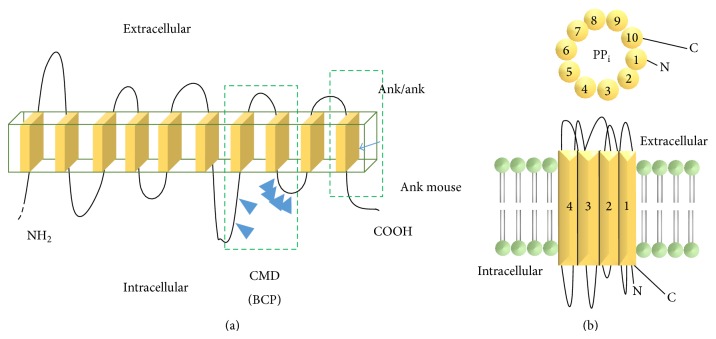
Schematic diagram of the structure of ANK protein. ANK protein is a type II transmembrane protein that spans the cell membrane with 10 helices. Most of the mutations responsible for CMD in humans fall in the intracellular sequence between 7 and 9 helix. Nonsense natural mutation in ANK mice locates toward the C-terminus on the 10th helix (a). The ANK protein works as a transporter that exports PP_i_ from inside out of the cell (b).

**Figure 11:**
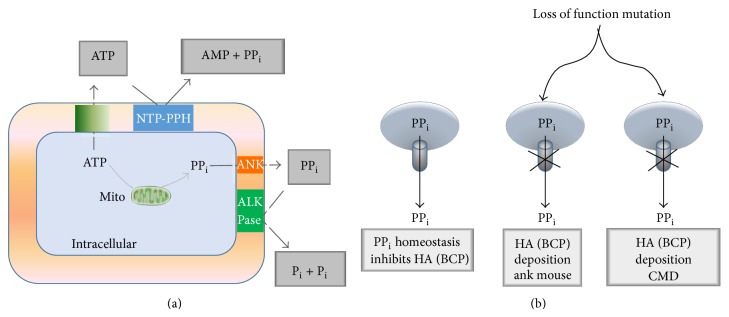
Schematic diagram of the pathogenesis of CMD. PP_i_ is generated from ATP hydrolysis intracellular by the mitochondria (Mito) or extracellular by the transmembrane enzyme nucleoside triphosphate pyrophosphohydrolase (NTP-PPH). PP_i_ generated intracellular is exported by ANK transporter to the extracellular one and is hydrolysed into two P_i_ by alkaline phosphatase (ALP) (a). Loss of function mutation in ANK leads to accumulation of PP_i_ intracellular. Absence of extracellular PP_i_ results in excessive bone formation due to increased deposition of bone minerals; hydroxyapatite (HA) crystals made of basic calcium phosphate (BCP), responsible for CMD phenotype in humans (b).

**Figure 12:**
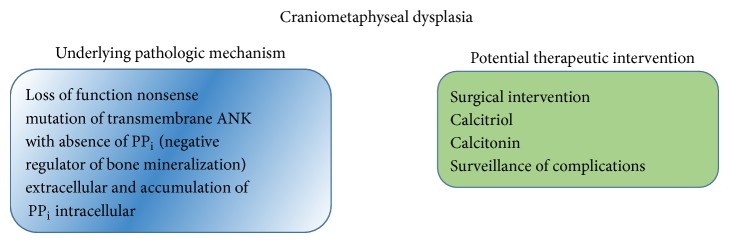
CMD pathogenesis and potential therapeutic interventions.

*Pathogenesis of CMD*. All CMD cases with known molecular diagnosis have so far been linked to ankh nonsense mutations on chromosome 6 that underlie increased intracellular and decreased extracellular pyrophosphates (PP_i_) [82, 86, 88, 89]. Recent studies of CMD also point to the role of PP_i_ in the regulation of the bone modeling/remodeling process. The ANKH protein is type II transmembrane with 10–12 helices, spanning the outer cell membrane, and is associated with PP_i_ efflux (Figure 10). Most of the ankh mutations are located in cytoplasmic domains close to the C-terminus [82, 86]. PP_i_ is a major inhibitor of physiologic, pathologic tissue calcification and bone mineralization. Intracellular PP_i_ is generated and stored largely in mitochondria, but it is also detected in endoplasmic reticulum and Golgi [90–94]. The extracellular PP_i_ concentration in the skeletal tissue is determined by several types of cell membrane proteins: ectoenzyme PC1, which generates PP_i_ from ATP, tissue nonspecific alkaline phosphatase (TNAP), which hydrolyzes PP_i_ into two inorganic phosphates (P_i_), and ANKH, which is involved in PP_i_ efflux (Figure 11). While the functional role of intracellular PP_i_ in mammalian cells remains elusive, extracellular PP_i_ has been extensively studied for its inhibitory role in tissue calcification. Extracellular PP_i_ directly binds to the surface of basic calcium phosphate hydroxyapatites and interferes with propagation of crystal formation, contributing to the formation of poorly ordered bone crystal structure [95, 96]. In addition, exogenous PP_i_ at micromolar concentrations stimulates the expression of osteopontin, which is a negative regulator of mineralization, and inhibits the enzymatic activity of tissue nonspecific alkaline phosphatase (ALP) in osteoblast cultures [96, 97]. Thus, a decrease in extracellular PP_i_ may hinder normal bone remodeling, for instance, by inhibiting osteoclast differentiation or activity. In support of this notion, bone marrow-derived monocytes (BMMs) from a CMD knock-in mouse (p.Phe377del in ank) poorly differentiated to osteoclasts in cultures, compared to those from wild type mice [98]. Consistent with the mouse data, the number of bone marrow-derived osteoclast-like cells from a CMD patient was only 40% of a normal individual, and they lacked osteoclast-specific vacuolar proton pump and the ability to absorb a dentin slice [99]. The ANKH protein may have also other, unknown functions (Figure 12).

*Treatment*. Therapeutic intervention consists primarily of surgery aiming to decompress the nerve canal and/or narrowed foramen magnum. Excessive bony overgrowth of facial bone, forehead, and cranial regions can be contoured; however, bone regrowth is common. For severe complications, surgery is conservative to relieve severe symptoms caused by cranial nerve compression. Surveillance of patients is crucial as bone growth continues throughout life, and the patients will require regular neurologic evaluation, hearing assessment, and ophthalmologic examination for early diagnosis and management of complications. Therapeutic trial with calcitriol that stimulate bone resorption, with low calcium diet, has been reported to improve facial paralysis but has no effect on metaphyseal deformity [100]. Trial with calcitonin has been thought to be effective due to its inhibitory effect on bone turnover which is inefficient in treating hyperplasia of craniofacial bones in persons with CMD [101].

## 9. Achondroplasia 

Achondroplasia (chondrodysplasias) is a human bone genetic disorder of the growth plate and is the most common form of dwarfism [102]. Achondroplasia is caused by AD mutations of the transmembrane receptor fibroblast growth factor receptor 3 (FGFR3), an important regulator of linear bone growth [103, 104]. Achondroplasia has an incidence rate of one in 20,000 live births; and it results from a spontaneous heterogeneous mutation to nonachondroplastic parents in an estimated 80% of cases [102, 105].

*Clinical Diagnosis*. Achondroplasia is most likely recognized at birth because of its characteristic clinical and radiographic features. Achondroplasia in newborn infants classically presents with disproportionate shortening of the limbs, a long and narrow trunk, a large head with frontal bossing, and a hypoplastic midface. The hands are short and broad, often displaying a three-pronged (trident) configuration. Moreover, many joints show hyperextensibility and infants are often hypotonic. Skeletal x-rays of the newborn infant reveal characteristic abnormalities that include shortening of the long bones of the limbs, particularly the proximal bones, with metaphyseal irregularities. The pelvis is abnormal with small and square iliac wings. The cranium is large with a prominent forehead with midface hypoplasia.

*Pathogenesis*. Achondroplasia is an AD genetic disorder, where it is linked to mutations of FGFR3 on the distal short arm of chromosome 4 [106, 107]. Patients with achondroplasia have nonsense genetic mutation in FGFR3 with glycine to arginine substitution at position 380 (G380R), in the transmembrane domain of the receptor [105]. However, additional FGFR3 mutations have been detected in hypochondroplasia, achondroplasia with developmental delay, and acanthosis nigricans, Muenke craniosynostosis and Crouzon syndrome with acanthosis nigricans [102, 105, 108]. However, the diagnosis can be established from DNA mutational analysis. Mutational diagnosis can also be used for prenatal especially in couples at risk of having baby with homozygous achondroplasia.

FGFR3 mutations in mice have identified the function of FGFR3 in skeletal development and postnatal bone formation. The global knockout of FGFR3 generated large mice with longer than normal limb bones [109, 110]. However, knocking in FGFR3 with achondroplasia mutation in cartilage of transgenic mice produced a small mouse with short bones, a phenotype similar to those seen in human achondroplasia [111]. Collectively, these observations established the fact that FGFR3 is an important negative regulator of endochondral bone formation and that the mutations cause a constitutive activation of FGFR3, resulting in achondroplasia and related dwarfing phenotype.

**Figure 13:**
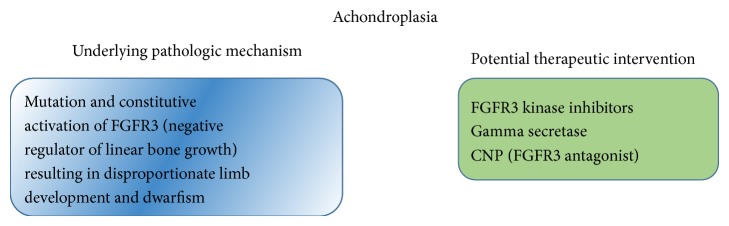
Achondroplasia pathogenesis and potential therapeutic interventions.

*Treatment*. A number of therapeutic approaches have been attempted to reduce excessive activation of FGFR3 as possible treatments to normalize bone growth in achondroplasia. They include strategies to interfere with FGFR3 synthesis, block its activation, inhibit its tyrosine kinase activity, promote its degradation, and antagonize its downstream signals. These treatment modalities include FGFR3 kinase inhibitors and gamma-secretase that modulate FGFR3 cleavage and nuclear function. Another valuable therapeutic candidate in the treatment of achondroplasia is CNP that works as an antagonist to FGFR3 signal. A previous study revealed that transgenic mice overexpressing brain natriuretic peptide (BNP) in the liver exhibited postnatal skeletal overgrowth with elongation of long bone growth plates [112]. Another study showed that CNP is more potent than BNP in stimulating bone growth by using tibial organ culture experiments, suggesting that CNP was the physiological ligand in growing bones [113]. Global knockout of CNP in mice showed severe postnatal dwarfism that was rescued after crossing with mice overexpressing CNP from a transgene driven by the cartilage-specific* COL2A1* promoter [114]. These results confirmed the stimulatory effects of CNP on endochondral ossification* in vivo*. To explore the beneficial effects of CNP in treating achondroplasia, mice overexpressing CNP in cartilage were crossed with mice displaying an achondroplastic phenotype due to overexpression mutation of FGFR3 [115]. Interestingly, the skeletal growth defect in the achondroplastic mice was corrected by the local overexpression of CNP. The results suggested that CNP antagonizes the active FGFR3 possibly by inhibition of MAPK-mediated FGFR3 signaling (Figure 13).

## 10. Hypophosphatasia

Hypophosphatasia (HPP) is an inherited metabolic bone disorder [116], caused by genetic loss of function mutation(s) of tissue-nonspecific alkaline phosphatase (*TNSALP*) [117]. Therefore, the high extracellular inorganic pyrophosphate (PP_i_), a TNSALP substrate with inhibiting effects on mineralization accumulates, leads to subnormal extracellular concentrations of calcium and P_i_ that result in rickets or osteomalacia [117]. HPP is an exception where the circulating levels are usually normal or elevated [118]. Despite the high levels of TNSALP in bone, cartilage, liver, and kidney in healthy individuals, HPP appears to disrupt only ALP in “hard tissues” directly [118]. HPP is characterized by a wide-ranging expressivity that ranges from death* in utero* with almost an unmineralized skeleton to difficulties with adult teeth without skeletal disease. Five major forms of HPP have been identified based on clinical diagnosis. The age at diagnosis of skeletal disease determines the perinatal, infantile, childhood, and adult types of HPP [118]. Individuals without skeletal findings but dental features only are said to have “odonto-HPP” [118]. Autosomal recessive (AR) and autosomal dominant (AD) inheritance partially explain the remarkable range of HPP severity [117]. Perinatal and infantile HPP cases are inherited as an AR trait, whereas the more mild forms may reflect AR or AD inheritance [117, 119]. To date, 224 different defects in* TNSALP* (80% missense mutation) have been identified in HPP that explain the extreme range of severity of this disorder. The prognoses for these five major forms of HPP are determined by the skeletal complications. Typically, the earlier the signs and symptoms, the worse the outcome [118].

*Pathogenesis of HPP*. The bone disease is due to missense mutation of TNSALP with structural defects. Many* TNSALP* mutations responsible for HPP change a conserved amino acid in the mammalian TNSALPs [120]. Some mutations disturb the catalytic pocket or the structural binding site for metal ligand; others compromise dimer formation [118, 120]. Moreover, some mutations impair the intracellular movement of TNSALP [120]. TNSALP deficient mice have confirmed insight from HPP patients and showed reduced longitudinal growth and delayed epiphyseal ossification, accompanied by disturbance in the mineralization pattern. It is concluded that ablation of TNALP results in hypomineralization of the skeleton with sever disordered mineralized matrix architecture [121].

**Figure 14:**
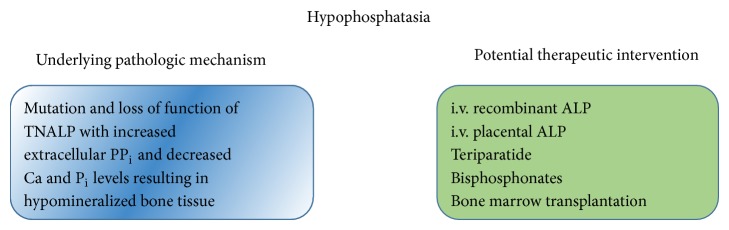
Hypophosphatasia pathogenesis and potential therapeutic interventions.

*Prognosis*. Perinatal HPP is always fatal. Infantile HPP often features clinical and radiographic deterioration with approximately 50% of babies dying from respiratory compromise [122, 123]. Childhood HPP may get improved after fusion of the growth plates. Skeletal problems are likely to return in adulthood [124]. Adult HPP causes recurrent and long lasting orthopedic difficulties (Figure 14).

*Treatment*. There is no established therapeutic protocol of HPP, although several approaches have been attempted, including intravenous infusions of soluble recombinant ALP [125], bone marrow transplantation [123], and teriparatide administration [124]. Bisphosphonates (derivatives of PP_i_) could be ineffective or pose further problems [118]. It has been reported that plasma and urine PP_i_ decrease after placental ALP correction of the hypophosphatasia in pregnant carriers of HPP [118] and i.v. injection of purified placental ALP was used to correct hypophosphatasemia in a severely affected infant, but there was no clinical or radiographic improvement. These negative results suggested the greater tissue need for ALP, or perhaps ALP must be bound to plasma membranes for therapeutic efficacy.

## 11. Conclusion 

There is yet a large scale of work needed to be done towards the discovery of new therapeutic methods of rare genetic bone disorders. The elucidation of disease mechanisms will provide the first step. Several potential therapeutic interventions have been proposed; however, implementation of these therapeutic strategies will take time. The disease mechanism of Gorham-Stout disease, melorheostosis, and multiple hereditary exostosis still needs to be fully elucidated. The development of inhibitors of the ACVR1/ALK2 pathway seems to show promise as a possible therapeutic intervention for FOP. The use of bisphosphonates and IL-6 inhibitors may be useful in the treatment of fibrous dysplasia, but further studies are needed. A viable cell therapy, bisphosphonate polytherapy, and HGH may have potential to avert the pathology in osteogenesis imperfecta, but more research is needed to prove therapeutic benefit.

The need for cures to these rare bone disorders has never been more pressing, given the increasing number of afflicted individuals living across the globe. Furthermore, potential cures for these rare bone disorders may also impact the management of more common bone diseases that display the same basic mechanisms such as heterotrophic ossification. Thus, research in the upcoming years will show that viable therapies of rare bone disorders might be in the horizons.
